# B7-H1 antibodies lose antitumor activity due to activation of p38 MAPK that leads to apoptosis of tumor-reactive CD8^+^ T cells

**DOI:** 10.1038/srep36722

**Published:** 2016-11-08

**Authors:** Xin Liu, Xiaosheng Wu, Siyu Cao, Susan M. Harrington, Peng Yin, Aaron S. Mansfield, Haidong Dong

**Affiliations:** 1 Department of Urology, College of Medicine, Mayo Clinic, Rochester, Minnesota 55905, USA; 2 Division of Hematology, College of Medicine, Mayo Clinic, Rochester, Minnesota 55905, USA; 3 Department of Immunology, College of Medicine, Mayo Clinic, Rochester, Minnesota 55905, USA; 4 Division of Medical Oncology, College of Medicine, Mayo Clinic, Rochester, Minnesota 55905, USA.

## Abstract

B7-H1 (aka PD-L1) blocking antibodies have been used in treatment of human cancers through blocking B7-H1 expressed by tumor cells; however, their impact on B7-H1 expressing tumor-reactive CD8^+^ T cells is still unknown. Here, we report that tumor-reactive CD8^+^ T cells expressing B7-H1 are functional effector cells. In contrast to normal B7-H1 blocking antibody, B7-H1 antibodies capable of activating p38 MAPK lose their antitumor activity by deleting B7-H1^+^ tumor-reactive CD8^+^ T cells via p38 MAPK pathway. B7-H1 deficiency or engagement with certain antibody results in more activation of p38 MAPK that leads to T cell apoptosis. DNA-PKcs is a new intracellular partner of B7-H1 in the cytoplasm of activated CD8^+^ T cells. B7-H1 suppresses p38 MAPK activation by sequestering DNA-PKcs in order to preserve T cell survival. Our findings provide a new mechanism of action of B7-H1 in T cells and have clinical implications in cancer immunotherapy when anti-B7-H1 (PD-L1) antibody is applied.

B7-H1 (also named PD-L1 or CD274) expressed by human tumor cells and its receptor PD-1 expressed on effector T cells constitute an important immune regulatory pathway in restraining antitumor function of T cells^1^,^2^,^3^. Antibodies capable of blocking the binding of B7-H1 and PD-1 have been used in clinical trials in treatment of various cancers in humans^4^,^5^, and recently one anti-PD-L1 antibody (atezolizumab) has been approved to treat bladder cancers. However, only a subset of patients respond completely or partially to B7-H1 blockade therapy^6^. To improve the efficacy of anti-B7-H1 blocking antibodies, it is crucial to understand the mode of action of anti-B7-H1 antibodies in the context of tumor-T cell interactions. Although B7-H1 expressing tumor cells are the intended target of anti-B7-H1 antibody therapy, tumor-infiltrating lymphocytes (TILs) is likely targeted by the same therapy since they have also been shown to express B7-H1. Indeed, the level of B7-H1 positive TILs have recently been found to be correlated with responses to anti-PD-1 therapy^5^,^6^, suggesting B7-H1 expressing lymphocytes within tumor tissues may determine the final outcome of anti-B7-H1 therapy in human cancers. However, very few studies have addressed the function of B7-H1 positive tumor-reactive CD8^+^ T cells (T_TR_ cells) within tumor tissues and the direct impact of anti-B7-H1 antibodies on those cells. Some preclinical studies have shown that not all B7-H1 blocking antibodies lead to improved CD8^+^ T cell responses *in vivo*^7^,^8^, and the underlying mechanisms of anti-B7-H1 blocking antibody action is largely unknown. It is possible that B7-H1 blocking antibodies are capable of triggering B7-H1′s intracellular signaling pathway in T_TR_ cells in addition to their role in blocking B7-H1/PD-1 interactions^9^. Once initiated by B7-H1 antibody, the intracellular signaling of B7-H1 may synergize with or antagonize the ultimate antitumor function of T_TR_ cells. To address these questions, we evaluated the therapeutic effects of anti-B7-H1 blocking antibodies in context of their effects on B7-H1 expressing T_TR_ cells. We show that in contrast to normal B7-H1 blocking antibody, B7-H1 antibodies capable of activating p38 MAPK lose their antitumor activity by deleting B7-H1^+^ tumor-reactive CD8^+^ T cells via p38 MAPK pathway. B7-H1 deficiency or engagement with certain antibody results in more activation of p38 MAPK that leads to T cell apoptosis. Mechanistic studies indicate that DNA-PKcs is a new intracellular partner of B7-H1 in activated CD8^+^ T cells that need B7-H1 to suppress p38 MAPK activation by sequestering DNA-PKcs in order to preserve T cell survival.

## Results and Discussion

### B7-H1 expression identifies effector CTLs within tumor tissues

**Figure 1:**
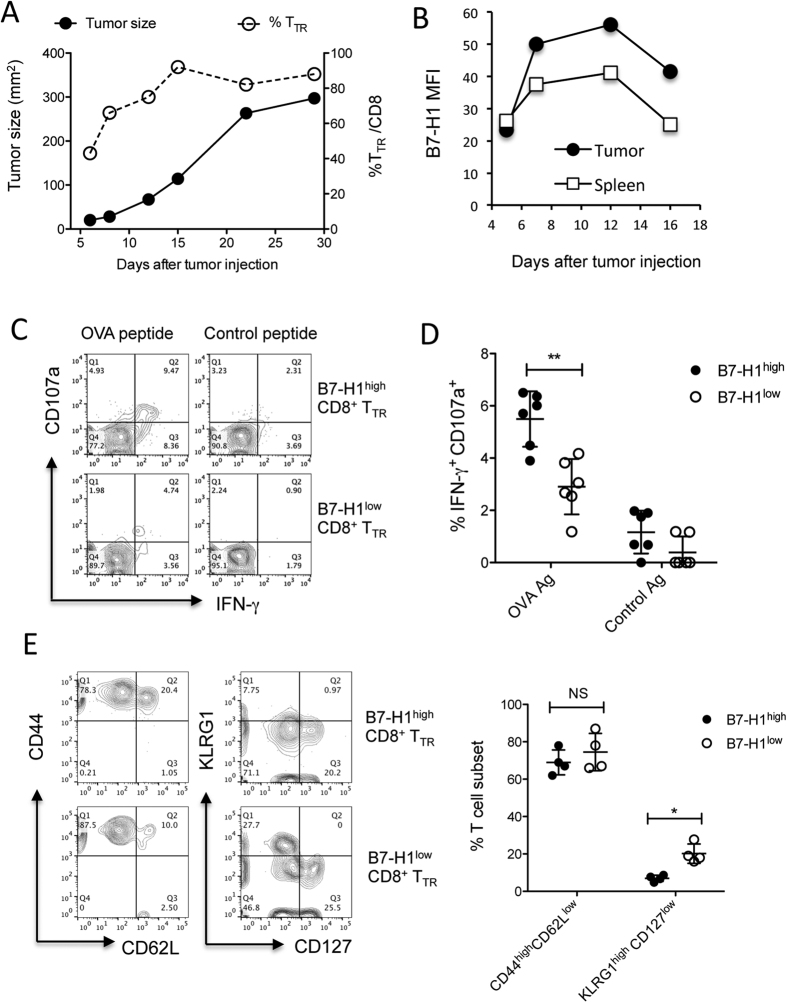
B7-H1 expressed by CD8^+^ T cells identifies effector T_TR_ cells in tumor tissues. (**A**) Frequency of tumor-reactive (PD-1^+^CD11a^high^) CD8^+^ T_TR_ cells were identified within B16-OVA tumor tissues (dash line) along with B16-OVA tumor growth (solid line, average size of 5 mice). (**B**) Kinetics of B7-H1 levels measured by flow cytometry in CD8^+^ T_TR_ cells from tumor tissues and spleen of B16-OVA tumor bearing mice, or from CD8^+^ T cells of naïve mice. MFI: mean fluorescence intensity. (**C**,**D**) CTL function among B7-H1^high^ and B7-H1^low^ CD8^+^ T_TR_ cells was analyzed by measuring degranulation (CD107a) and IFN-γ production following a brief *ex vivo* re-stimulation with OVA antigen peptides or control peptide for 5 hours, **P < 0.01 (mean ± s.d., Mann-Whitney test). (**E**) Phenotype of B7-H1^high^ and B7-H1^low^ CD8^+^ T_TR_ cells infiltrating tumors. B7-H1^low^ CD8^+^ T cells exhibited more short-lived effector cell phenotype (KLRG-1^high^ CD127^low^ compared to B7-H1^high^ CD8^+^ T cells, *P < 0.05 (mean ± s.d., n = 4, Mann-Whitney test). NS, non- significant.

Since B7-H1 expression on TILs has a predictive value in anti-B7-H1 therapy response^5^,^6^, we first examined the expression kinetics of B7-H1 on T_TR_ cells within growing tumors. Using PD-1^+^CD11a^high^ expression as a surrogate marker to define T_TR_ cells^10^,^11^, we found that the number of T_TR_ cells gradually increased and peaked around day 15 after tumor seeding in mice (Fig. 1A). Furthermore, the level of B7-H1 expression on T_TR_ cells also gradually increased up to day 12 and declined thereafter (Fig. 1B). High B7-H1 expression on T_TR_ cells is associated with better effector function of T_TR_ cells, i.e. B7-H1^high^ CD8^+^ T_TR_ cells produced more IFN-γ and expressed more CD107a (a degranulation marker) upon *ex vivo* re-stimulation with surrogate tumor antigen OVA peptides (Fig. 1C,D). Phenotype analysis of B7-H1^high^ and B7-H1^low^ CD8^+^ T cells within tumors showed a similar effector memory (CD44^high^ CD62L^low^) phenotype (Fig. 1E), while B7-H1^low^ CD8^+^ T cells exhibited more short-lived effector cell phenotype (KLRG-1^high^ CD127^low^)^12^ than B7-H1^high^ CD8^+^ T cells (P < 0.05). Therefore, lower B7-H1 expression may be a unique phenotype of short-lived effector cells. This observation is consistent with our previous finding that B7-H1 is required by activated effector T cells to survive during the contraction phase^13^. Our results explain why B7-H1 positive TILs are a predictive marker for responders to anti-PD-1 or anti-B7-H1 therapy, because B7-H1 expression identifies the pre-existing CD8^+^ effector T cells capable of eliminating tumors.

### B7-H1 antibody capable of activating p38 MAPK enhances CD8^+^ T cell apoptosis

**Figure 2:**
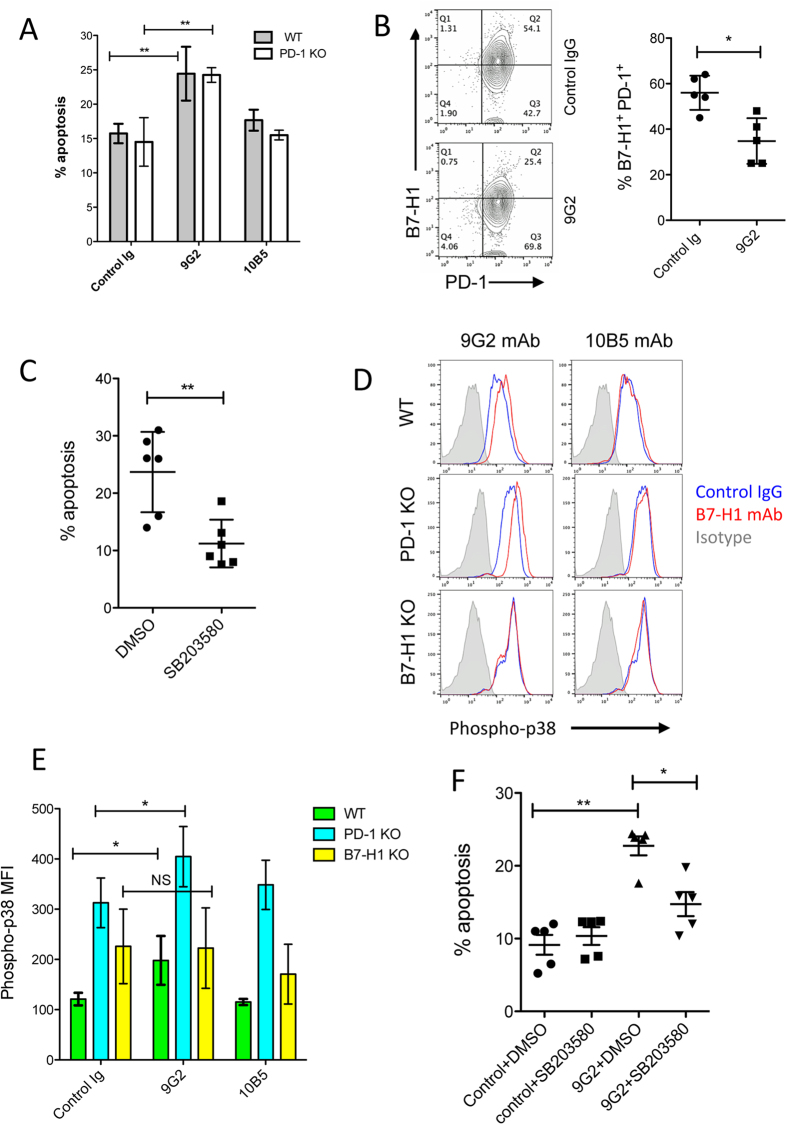
B7-H1 antibody enhances CD8^+^ T cell apoptosis via activation of p38 MAPK. (**A**) Anti-B7-H1 9G2 mAb, but not 10B5 mAb, increased apoptosis of activated CD8^+^ T cells isolated from WT and PD-1 KO mice (n = 3). **P < 0.01 (mean ± s.d., analyzed by two-way ANOVA). (**B**) Depletion of B7-H1^+^ PD-1^+^ CD8^+^ T_TR_ cells by 9G2 mAb within tumor tissues. On day 3 of last 9G2 mAb or control IgG treatment, TILs were isolated and stained for CD8, CD11a, PD-1 and B7-H1. Dot plot graphs show cells gated on CD11a^high^ CD8^+^ T cells. *P < 0.05 (mean ± s.d., Mann-Whitney test). (**C**) Inhibitors of p38 MAPK decrease apoptosis of activated CD8^+^ T cells. Pre-activated mouse CD8^+^ T cells were incubated with SB203580 (an inhibitor of p38 MAPK at 10 μM) or solvent control DMSO for 48 hours. The percentages of apoptotic T cells (TMRE^low^ Annexin V^+^) were shown. **P < 0.01 (mean ± SD, Mann-Whitney test). (**D**) B7-H1 mAb in activation of p38 MAPK in wild type, B7-H1 KO or PD-1 KO CD8^+^ T cells. Histograms show the levels of phospho-p38 MAPK in CD8^+^ T cells in culture with 9G2 or 10B5 mAb (red lines) or control IgG (blue lines). Isotype curves are cells stained with control rabbit IgG as an isotype control for phosphor-p38 MAPK staining. (**E**) 9G2 mAb, but not 10B5 mAb, increased the levels (MFI) of phosphorylated p38 MAPK (phospho-p38) in activated WT and PD-1 KO, but not B7-H1 KO, CD8^+^ T cells. *P < 0.05 (mean ± s.d., analyzed by one-way ANOVA, n = 6). NS, non- significant. One of three independent experiments is shown. (**F**) Inhibitor of p38 MAPK (SB203580) reduced apoptosis of CD8^+^ T cells induced by 9G2 mAb. Pre-activated CD8^+^ T cells were incubated with plate-coated 9G2 mAb or control IgG for 48 hours with DMSO or SB203580 (10 μM) followed with staining with TMRE and Annexin V. Apoptotic T cells were identified by their phenotype of TMRE^low^ and Annexin V^+^. *P < 0.05 and **P < 0.01 (mean ± s.e.m., n = 5, two-tailed paired t test).

We previously reported that B7-H1 expressed by activated CD8^+^ T cells is required for T cell survival^13^, and ligation of B7-H1 by certain antibodies causes more apoptosis in T cells^9^. To test whether B7-H1 blocking antibodies have the ability to induce apoptosis of T cells, we selected B7-H1 monoclonal antibodies (mAb), clone 10B5^14^ and 9G2^15^, since both of them have been used widely in animal models and have a defined B7-H1/PD-1 blocking function. The results of Fig. 2A show that engagement of pre-activated CD8^+^ T cells with 9G2 but not 10B5 mAb, significantly increased T cell apoptosis *in vitro* (P < 0.05), and this effect was PD-1 independent as 9G2 mAb induced a similar degree of apoptosis in PD-1 KO CD8^+^ T cells as in the wild type CD8^+^ T cells. Consistent with its role in promoting T cell apoptosis *in vitro*, treatment with 9G2 mAb indeed resulted in a significant reduction of B7-H1^+^ PD-1^+^ CD8^+^ T cells *in vivo* within tumor tissues (Fig. 2B).

To explore the mechanism underlying B7-H1 antibody–induced apoptosis, we measured the phosphorylation of p38 MAPK in activated CD8^+^ T cells. The reason to examine p38 MAPK activation is that CD8^+^ T cells maintain a higher level (2–3 fold) of p38 MAPK activity than CD4^+^ T cells, and activation of p38 MAPK causes a selective loss of CD8^+^ T cells in peripheral lymphoid organs^16^. The loss of CD8^+^ T cells is attributed to increased apoptosis mediated by caspases following p38 MAPK activation^17^. To confirm the role of p38 MAPK in CD8^+^ T cell apoptosis, we examined whether p38 MAPK inhibitor SB203580 is able to reduce apoptosis of CD8^+^ T cells. In an activation-induced T cell death model, the p38 MAPK inhibitor SB203580 dramatically reduced the apoptosis of activated CD8^+^ T cells *in vitro* (Fig. 2C), suggesting activation of p38 MAPK indeed leads to more CD8^+^ T cell apoptosis.

Next, we tested whether B7-H1 antibody-induced T cell apoptosis is due to the activation (phosphorylation) of p38 MAPK. Consistent with its ability in enhancing T cell apoptosis, the 9G2 mAb, but not 10B5 mAb (or to a much lesser extent), significantly increased the phosphorylation of p38 MAPK in wild type CD8^+^ T cells (P < 0.05, Fig. 2D,E). Of note, 9G2 mAb increased p38 MAPK phosphorylation in PD-1 KO CD8^+^ T cells, but not in B7-H1 KO CD8^+^ T cells (P < 0.05, Fig. 2E), suggesting 9G2 mAb-induced activation of p38 MAPK requires B7-H1 but not PD-1. In contrast, 10B5 mAb did not increase p38 MAPK activation in WT, PD-1 KO nor B7-H1 KO CD8^+^ T cells (Fig. 2D,E). We then tested whether 9G2 antibody-induced CD8^+^ T cell apoptosis is dependent on the activation of p38 MAPK or not. The results of Fig. 2F show that p38 MAPK inhibitor SB203580 significantly abolished 9G2-induced T cell apoptosis (P < 0.05), suggesting activation of p38 MAPK is a mechanism of T cell apoptosis induced by 9G2 mAb. Taken together, B7-H1 antibody capable of activating p38 MAPK has the ability to enhance the apoptosis of CD8^+^ T cells.

In addition to our murine system, we screened human B7-H1 mAbs for their potential capability of activating p38 MAPK in human CD8^+^ T cells. Among a panel of human B7-H1 mAbs, we found that H1A and 2.2B mAbs significantly increased the phosphorylation of p38 MAPK in activated human CD8^+^ T cells (Supplemental Fig. 1). Therefore, this new function of B7-H1 antibodies is not a species-specific feature. Interestingly, H1A and 5H1 mAbs are both IgG1 but have different binding sites, indicating that the epitope-binding site, but not the antibody isotype, largely determines the potential to induce activation of p38 MAPK.

### B7-H1 antibody capable of activating p38 MAPK in CD8^+^ T cells fails to suppress tumor growth

**Figure 3:**
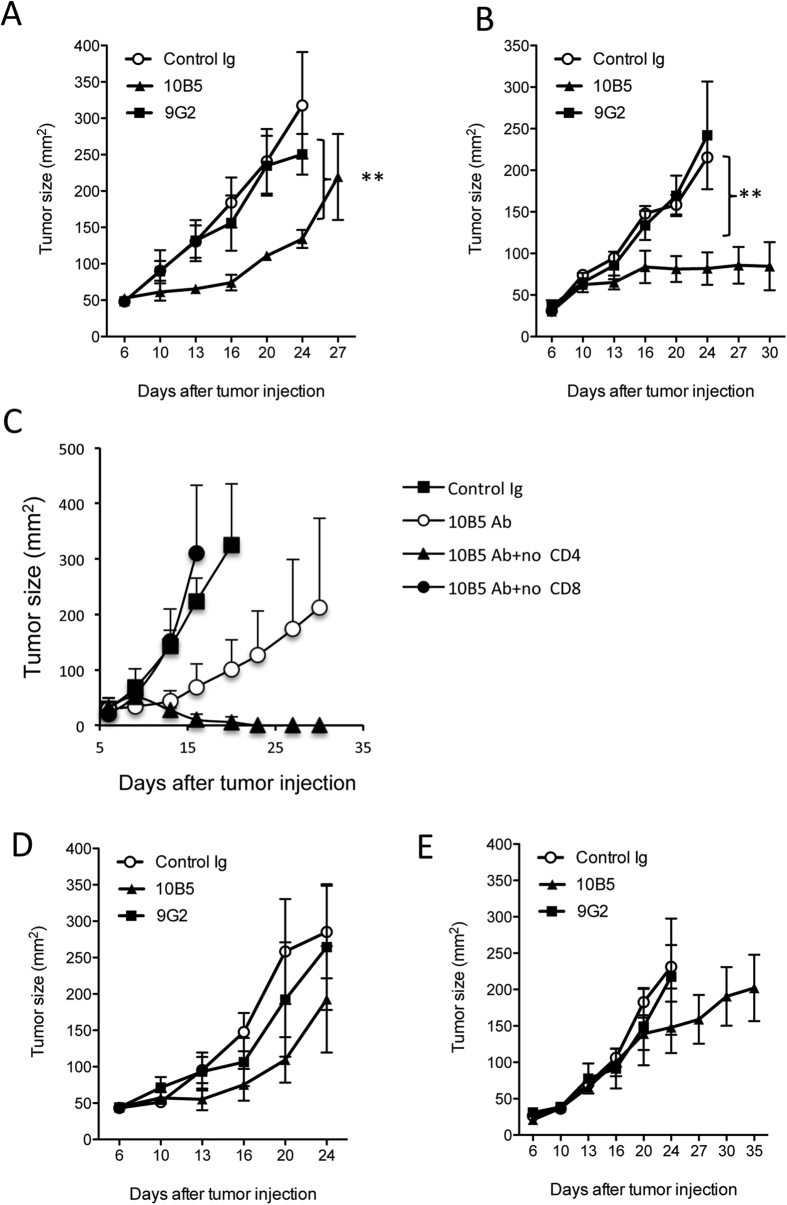
B7-H1 antibodies capable of activating p38 MAPK fail to suppress tumor growth. B16-OVA (**A**) or RENCA tumors (**B**) were treated with intratumoral injection with control IgG or anti-B7-H1 mAb (9G2 or 10B5, respectively) on day 6 or 7 after tumor injection at 20 μg for 5 doses at three-day interval. **P < 0.01 compared with control groups (mean ± s.d., five mice per group, two-way ANOVA). (**C**) Depletion of CD8^+^ T cells, but not CD4^+^ T cells, abolished antitumor function of 10B5 mAb. B16-OVA (**D**) or RENCA tumors (**E**) were treated with intra-peritoneal injection with control IgG or anti-B7-H1 mAb (9G2 or 10B5, respectively) on day 7 after tumor injection at 200 μg for 5 doses at three-day interval. No significant suppression of tumor growth was identified in both B7-H1 antibody-treated groups.

Having demonstrated the pro-apoptotic role of B7-H1 antibody capable of activating p38 MAPK in activated CD8^+^ T cells, we then evaluated the antitumor effect of this B7-H1 antibody (9G2). We treated B16-OVA tumors (a mouse melanoma model) with 9G2 mAb or 10B5 mAb by intra-tumor injection. Although both B7-H1 mAbs 10B5^14^ and 9G2^15^ are capable of blocking B7-H1/PD-1 binding, only 10B5 mAb significantly suppressed the tumor growth while 9G2 mAb showed little antitumor activity in comparison with control group (Fig. 3A). Similarly, we found treatment with 10B5 mAb effectively suppressed RENCA (a mouse renal cell carcinoma) growth in Balb/c mice while with 9G2 mAb exhibited no therapeutic benefit (Fig. 3B). To test whether the therapeutic effect of 10B5 mAb is mediated by CD8^+^ T cells^13^,^18^, we depleted CD8 or CD4 T cells prior to and during the treatment of 10B5 mAb. The results of Fig. 3C show that 10B5 mAb lost its therapeutic effects if CD8^+^ T cells, but not CD4^+^ T cells, were depleted in the host. Interestingly, depletion of CD4^+^ T cells significantly improved the therapeutic effects of 10B5 (Fig. 3C). It is likely that anti-CD4 mAb treatment augmented both the quantity and quality of tumor-specific CD8^+^ T-cell responses through the removal of CD4^+^ immunosuppressive cells^19^.

To test whether the route of injection would have any impact on the therapeutic effects of 9G2 or 10B5 mAbs, we performed intra-peritoneal injection of B7-H1 antibodies in treatment of established tumors. The results of Fig. 3D,E show that intra-peritoneal injection of 9G2 mAb did not significantly suppress tumor growth in either B16 or RENCA models, although 10B5 mAb suppressed tumor growth to some degree, suggesting the route of delivery of 9G2 would not change its function. The failure of 9G2 mAb in treatment of tumors is in line with its failure in promoting CD8^+^ T cell responses in infection models^7^,^8^. The underlying mechanism in these infection models could be due to 9G2′s capability in activation of p38 MAPK that needs future investigation. Taken together, our results indicate that B7-H1 antibodies capable of activating p38 MAPK lose their antitumor activity through deleting B7-H1^+^ effector CD8^+^ T cells.

### B7-H1 regulates p38 MAPK activation through association with DNA-PKcs

**Figure 4:**
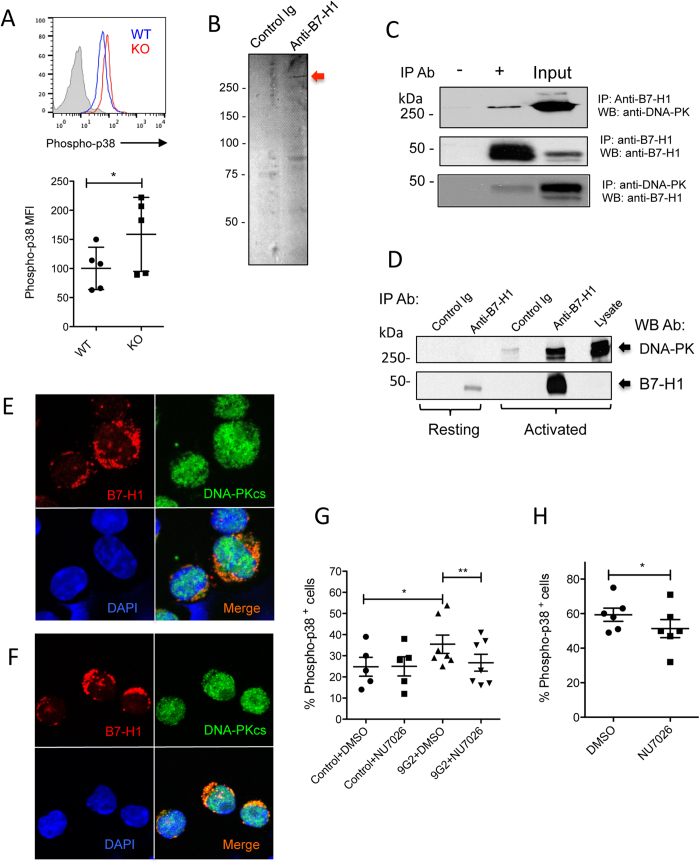
B7-H1 regulates p38 MAPK activation via association with DNA-PKcs. (**A**) Increased activation of p38 MAPK in B7-H1 KO CD8^+^ T cells. CD8^+^ T cells isolated for WT or B7-H1 KO mice were activated with anti-CD3/CD28 beads for 48 hours followed with analysis of phosphorylation of p38 MAPK by flow cytometry. *P < 0.05 (mean ± s.d., gender and age paired 2-tailed t test). (**B**) Anti-B7-H1 Ab, but not control Ab, co-precipitated a protein band in Karpas 299 cells (arrow). (**C**) Immunoprecipitation (IP) and Western blot (WB) identified an association of B7-H1 with DNA-PKcs in Karpas 299 cells. Whole cell lysate is used as input. The association of B7-H1 with DNA-PKcs was detected by Western blotting using B7-H1 mAb or anti-DNA-PKcs Ab. (**D**) B7-H1 and DNA-PKcs association was identified in human primary T cells activated by PHA or resting for 48 hours. IP antibody: B7-H1 mAb (5H1) or control IgG. Membranes were probed with anti-DNA-PK (H163) or anti-B7-H1 mAb (H1A). Data of D and E are representative cropped blots from the full-length blots that are included in the supplemental Figs 3 and 4. The association of B7-H1 and DNA-PKcs was identified in the cytoplasm of wild type (**E**) and PD-1 KO (**F**) activated CD8^+^ T cells. (**G**) DNA-PKcs inhibitor (NU7026 at 1 μM) decreased phosphorylation of p38 MAPK in activated wild type CD8^+^ T cells stimulated with 9G2 antibody (n = 7), but not in wild type CD8^+^ T cells treated with control IgG (n = 5). (**H**) NU7026 suppressed phosphorylation of p38 MAPK in B7-H1 KO activated CD8^+^ T cells (n = 6). *P < 0.05 and **P < 0.01 (mean ± s.e.m., two-tailed paired t test).

The fact that ligation of B7-H1 by certain antibodies leads to activation of p38 MAPK prompted us to examine whether B7-H1 has a T cell intrinsic function in regulation of p38 MAPK. To test that, we measured the phosphorylation of p38 MAPK in activated CD8^+^ T cells isolated from WT and B7-H1 KO mice. The results of Fig. 4A show that p38 MAPK activation increased in B7-H1 KO CD8^+^ T cells compared to B7-H1 WT CD8^+^ T cells, suggesting the presence of B7-H1 may intrinsically suppress the activation of p38 MAPK in CD8^+^ T cells. Consistent with our previous observation that B7-H1 KO CD8^+^ T cells failed to reject tumors *in vivo*^13^, the pro-survival function of B7-H1 in CD8^+^ T cells may be linked with its suppression of p38 MAPK activation.

Since p38 MAPK activation and T cell apoptosis induced by B7-H1 ligation with 9G2 mAb is independent of PD-1 (Fig. 2A,E), we assumed that this new function of B7-H1 is mediated by other intracellular signaling molecules that regulate p38 MAPK activation. To identify a potential intracellular, functional partner of B7-H1, we performed immunoprecipitation (IP) using anti-B7-H1 antibody (clone 5H1). We identified a 450 kDa protein band that co-precipitated with B7-H1 from human T lymphoma cell line Karpas 299 that constitutively expresses B7-H1 (Fig. 4B). Mass spectrometry analyses indicated that the most abundant protein in the 450 kDa band is the DNA-dependent protein kinase, catalytic subunit (DNA-PKcs). To further validate an interaction between B7-H1 and DNA-PKcs, we performed IP and Western assays. The results of Fig. 4C shows that DNA-PKcs and B7-H1 in Karpas 299 were mutually pulled down by their respective antibodies^20^. Significantly, an association of DNA-PKcs and B7-H1 was detected in activated (known to up-regulate B7-H1 expression) but not resting human T cells (Fig. 4D). To determine the location of the association of B7-H1 and DNA-PKcs in primary T cells, we stained B7-H1 and DNA-PKcs in activated and resting murine CD8^+^ T cells *in vitro*. The results of Fig. 4E show that B7-H1 co-localized with DNA-PKcs in the cytoplasm rather than in the nucleus where the dominant expression of DNA-PKcs was observed in activated T cells. Of note, the co-localization of B7-H1 and DNA-PKcs was also found in PD-1 KO activated murine T cells (Fig. 4F), suggesting the association of B7-H1 and DNA-PKcs is PD-1 independent. In contrast, only minimal co-localization of B7-H1 and DNA-PKcs were identified in the cytoplasm of resting T cells (wild type or PD-1 KO) that express less B7-H1 and DNA-PKcs (Supplemental Fig. 2). Our results suggest that B7-H1 regulates the activation of p38 MAPK in activated CD8^+^ T cells via association with DNA-PKcs.

Since DNA-PKcs increases among apoptotic T cells^21^,^22^, and DNA-PKcs is capable of regulating activation of MAPKs^23^,^24^, the association of B7-H1 and DNA-PKcs has a strong implication in regulating p38 MAPK in T cells. To test whether activity of DNA-PKcs is involved in p38 MAPK activation via association with B7-H1, we added a DNA-PKcs inhibitor NU7026 in culture of activated CD8^+^ T cells stimulated with or without 9G2 antibody. The results in Fig. 4G shows that NU7026 dramatically suppressed the activation (phosphorylation) of p38 MAPK induced by 9G2 mAb in activated CD8^+^ T cells, suggesting DNA-PKcs activity is required for the activation of p38 MAPK. It is not clear why NU7026 did not suppress p38 MAPK activation in activated CD8^+^ T cells treated with control IgG. It could be due to fewer “free” DNA-PKcs are present in the cytoplasm where most of DNA-PKcs are associated with B7-H1 (Fig. 4E,F). Once B7-H1 is ligated with 9G2 antibody or is knocked out, more “free” DNA-PKcs would be released to initiate p38 MAPK activation. It seems that only “free” DNA-PKcs is sensitive to the inhibition by NU7026, as NU7026 suppressed p38 MAPK activation in B7-H1 KO but not in wild type CD8^+^ T cells (Fig. 4 G,H). Future studies are warranted to examine how DNA-PKcs initiates the phosphorylation of p38 MAPK in CD8^+^ T cells. Given the common role of DNA-PKcs in DNA damage repairing in the nucleus, the association of DNA-PKcs with B7-H1 in the cytoplasm implies a new signaling function of DNA-PKcs in activated T cells. Targeting B7-H1/DNA-PKcs/p38 MAPK pathway could be a new approach to regulating CD8^+^ T cell function. Taken together, our results suggest that the association of B7-H1 and DNA-PKcs may sequester DNA-PKcs from its kinase function for phosphorylation of p38 MAPK in activated CD8^+^ T cells.

Since one B7-H1 (PD-L1) antibody (atezolizumab, Tecentriq) has been approved by the FDA for treatment of bladder cancers, more B7-H1 antibodies are being developed for the treatment of other cancers based on their blocking function of B7-H1 expressed by tumor cells. To that end, our results caution that some B7-H1 blocking mAbs may lose their *in vivo* antitumor activity by inducing T cell apoptosis through p38 MAPK activation. To avoid this scenario in cancer treatment with anti-B7-H1 antibody, B7-H1 blocking antibodies should be tested for their potential capability in activation of p38 MAPK or induction of apoptosis in activated CD8^+^ T cells. It seems that p38 MAPK inhibition may protect tumor-reactive T cells within tumors during treatment with anti-B7-H1 antibody, however, since p38 MAPK plays a dual role as a regulator of cell death in tumors, the use of p38 MAPK inhibitor should be carefully evaluated according to the types of stimulations in tumor cells or T cells^25^. Nevertheless, future studies are warranted to evaluate the combined benefits of p38 MAPK inhibition and B7-H1 antibody in cancer immunotherapy. On the other hand, while B7-H1 antibodies capable of activating p38 MAPK are not good candidates for treatment of cancers, their therapeutic potential in preservation of transplanted organs could be explored if they can selectively eliminate B7-H1^+^ allo-reactive T cells. This new possibility is highlighted by a recent report indicating B7-H1 is required by donor T cells to drive graft-versus-host disease lethality^26^.

In summary, our studies demonstrated the capability of certain B7-H1 antibody in activation of p38 MAPK in activated CD8^+^ T cells. B7-H1 antibodies with this particular capability lose their antitumor activity by deleting B7-H1^+^ tumor-reactive CD8^+^ T cells. DNA-PKcs is a new intracellular partner of B7-H1 in activated T cells. By sequestering DNA-PKcs, B7-H1 prevents activation of p38 MAPK in order to promote survival of CD8^+^ T cells. Our findings provide a new mechanism of action of B7-H1 in T cells and have clinical implications in cancer immunotherapy when anti-B7-H1 (PD-L1) antibody is applied.

## Materials and Methods

### Mice, cell lines and reagents

C57BL/6 mice and Balb/c were purchased from Jackson Lab (Bar Harbor, ME) or Taconic Farms (Germantown, NY) and maintained under pathogen-free conditions in the animal facility at Mayo Clinic Comparative Medicine Department. B7-H1 KO and PD-1 knockout (KO) C57BL/6 mice were provided by Dr. Lieping Chen (Yale University, New Haven, CT) with the permission of Dr. T. Honjo (Kyoto University, Japan) for PD-1 KO mice. B16-OVA murine melanoma cells were provided by Dr. R. Vile (Mayo Clinic, Rochester, MN). RENCA (mouse renal cell carcinoma) cell line was obtained from ATCC (Manassas, VA). Tumor cells were cultured in RPMI 1640 medium (Cellgro, Hendon, VA) with 10% FBS (Life Technologies, Carlsbad, CA), 1 U/ml penicillin, 1 μg/ml streptomycin, and 20 mM HEPES buffer (all from Mediatech, Manassas, VA). Anti-mouse B7-H1 mAb 9G2 (Clone 10.9G2) and its isotype control IgG were purchased from Bio X cell. Anti-mouse B7-H1 mAb (clone 10B5) was provided by Dr. Lieping Chen (Yale University). Studies were conducted in accordance with the National Institutes of Health guidelines for the proper use and care of animals in research and all experimental protocols were approved by the Institutional Animal Care and Use Committee of Mayo Clinic.

### Tumor treatment and tumor infiltrating lymphocyte analysis

C57BL/6 mice were injected subcutaneously (s.c.) with 5 × 10^5^ B16-OVA in the right flank. Perpendicular tumor diameters were measured using a digital caliper and tumor sizes were calculated as length × width. On day 6, when primary tumors were palpable, animals were randomly assigned to treatment groups. Briefly, B7-H1 or PD-1 antibody or control IgG at 20 μg was injected into tumor tissues in a volume of 50 μl of PBS daily for a total of three doses on days of 6, 9 and 12 post tumor injection. To deplete CD4^+^ or CD8^+^ T cells, anti-CD4 antibody (GK1.5, Bio X cell) or anti-CD8 antibody (2.43, Bio X cell) were injected i.p. at 500 μg on day 3, at 200 μg on days of 5, 8 and 11 post tumor injection. To isolate tumor-infiltrating lymphocytes (TILs) at indicated time points, tumor tissues were removed and incubated in digestion buffer (RPMI medium containing 5% fetal bovine serum, 0.02% Collagenase IV, 0.002% DNase I and 10 U/ml of Heparin) for 40 min folloed with isolation of lymphocytes. Tumor growth was evaluated every 2 to 3 days until days 35–40 when all mice were euthanized. In compliance with animal care guidelines, mice were euthanatized when either primary or secondary tumors reached 300 mm^2^.

### Flow cytometry analysis

Fluorochrome-conjugated Abs against mouse CD3, CD8, CD11a (M17/4), PD-1 (RMP1–30), B7-H1 (MIH5), CD107a and IFN-γ were purchased from BD Biosciences (Mountain View, CA), BioLegend (San Diego, CA), or eBioscience (San Diego, CA). To detect intracellular IFN-γ levels cells were incubated with GolgiPlug (BD Biosciences) for 4 hours prior to analysis. Cells were stained for surface antigens and then incubated in Fixation Buffer (BioLegend) for 20 min at room temperature, followed by permeabilization using Permeabilization Wash Buffer (BioLegend). After staining, cells were washed three times with washing buffer before analysis. At least 100,000 viable cells were live gated on FACSCailbur (BD Biosciences) instrumentation. Flow cytometry analysis was performed using FlowJo software (Tree Star, Ashland, OR).

### T cell pre-activation and apoptosis assay

Pre-activated CD8^+^ T cells were purified from spleen cells cultured with Con A (5 ug/ml, Sigma) for 48 hours. To inhibit the kinase activity of p38 MAPK, SB203580 (Cell Signaling #5633) was added at 10 μM in cell culture. Apoptosis of CD8^+^ T cells was analyzed by staining using Annexin V (BD Biosciences) and tetramethylrhodamine (TMRE; ethyl ester) (Invitrogen-Molecular Probes) for 10 min at room temperature followed with analysis with flow cytometry as we previously reported^13^.

### Measurement of phosphorylation of p38 MAPK

CD8^+^ T cells purified from spleens of wild type, PD-1 KO or B7-H1 KO mice, were incubated with plate-bound B7-H1 antibody (9G2 or 10B5) or control IgG for 48 hours in the presence of anti-CD3/CD28 beads (Dynabeads, Thermo Fisher Scientific). To inhibit the activity of DNA-PKcs, NU7026 (Selleckchem #S2893) was added at 1 μM in cell culture an hour before T cell activation. After incubation, cells are fixed in 4% formaldehyde for 10 min at 37 °C followed with permeabilization in ice-cold methanol for 30 min on ice. After washing, cells were blocked with Fc receptor blockers and incubated with rabbit mAb to phospho-p38 MAPK (Thr180/Tyr182) (Clone D3F9, Cell Signaling #4511) or Isotype control IgG for 1 hour at room temperature, followed with incubation with secondary anti-rabbit IgG (H + L), F(ab’)_2_ Fragment (Alexa Fluor^®^ 488 Conjugate) (Cell Signaling #4412) for 30 min at room temperature. For human CD8^+^ T cell analysis, T cells were incubated with anti-human B7-H1 antibody (clones: 5H1, H1A, MDX and 2.2B) in the presence of anti-CD3/CD28 beads for 24 hours before intracellular staining for phospho-p38 MAPK. The intracellular levels of p38 MAPK activation was analyzed by flow cytometry.

### Development of antibodies against human B7-H1

To generate monoclonal antibodies to human B7-H1, H1A, 2.2 B and B11, human 624mel cells were transfected with full-length human B7-H1 and injected (5 × 10^6^ cells per injection) intraperitoneally into Balb/c mice weekly for 6 weeks. Splenocytes from immunized mice were isolated and fused with A38 cells to form a hybridoma by standard techniques. H1A, 2.2 B and B11 hybridoma supernatants were screened by ELISA for reactivity against a recombinant human protein B7-H1, the extracellular domain of B7-H1 (amino acids 19–239) fused with human IgG Fc domain (R&D Systems), and to exclude their cross reactivity with irrelevant recombinant Fc fusion proteins of other B7 family members (R&D Systems).

### Immunoprecipitation (IP), Western blotting and Mass spectrometry

Immunoprecipitation of endogenous proteins that may be associated with B7-H1 was performed using B7-H1 positive Karpas-299 cells (a human T cell lymphoma cell line) purchased from American Type Culture Collection and propagated in complete media (RPMI, 10% FBS, 20 mmol/L HEPES, and penicillin/streptomycin). Cells were harvested in 1%NP40 lysis buffer containing 50 mM Tris-HCL pH 8.0, 150 mM NaCl, 5 mM EGTA, 5 mM EDTA, 30 mM MgCl2, 1.3% Beta-glycerophosphate, 1 mM DTT, 0.1 mM Na-Vanadate, 0.1 mM HaF, 0.4% p-nitrophenyl phosphate and protease inhibitors. Extracts were incubated overnight with protein G beads pre-coated with anti-B7-H1 mAb (5H1), control IgG or polyclonal anti-DNA-PKcs antibody (H163, Santa Cruz Biotechnology, Santa Cruz, CA). The proteins eluted from the protein G beads were resolved 5–7.5% SDS gel and detected by *Coomassie Blue* and Western blotting. Rabbit anti-DNA-PKcs antibody at 1:1000 dilution (H-163, Santa Cruz Biotechnology) and mouse anti-B7-H1 antibody at 1:500 dilution (H1A or B11, established at Dong lab) were used as the primary antibodies for Western blotting. Mass spectrometry analysis of protein bands were performed at Mayo Clinic Proteomics Research Core Facility.

### Immunofluorescence staining of T cells

Activated T cells were mounted to slides using a cytospin centrifuge (1000 rpm for 5 min) and fixed with 4% paraformaldehyde in phosphate-buffered saline (PBS) for 15 min at room temperature (RT). The cells were then permeabilized with 0.2% Triton X-100 in PBS for 10 min at RT and blocked with 3% nonfat milk or 5% BSA in PBS for at least 1 h at room temperature. Rabbit polyclonal antibody against DNA-PKcs (H-163, Santa Cruz Biotechnology) and rat monoclonal anti-B7-H1 antibody (MIH5, eBioscience) conjugated with Biotin were co-diluted (1:200) in 3% nonfat milk in PBS, and incubated with the cells for overnight at 4 °C followed by washing with 3% nonfat milk in PBS for 5 times. The cells were then further incubated with dylight 594 streptavidin (1:200, Vector), Alexa Fluor(R) 488 anti-rabbit IgG Fab2 (1:200, Cell Signaling) diluted in blocking buffer at room temperature for 1 hour. Finally, the slides were washed and mounted with DAPI (Vectashield). The stained slides were examined on a LSM780 laser-scanning microscope (Elvis, USA) with 100× objectives. Typically, 100 cells were examined in each experiment. Representative images were collected and processed using Adobe Photoshop (Adobe Systems).

### Statistical analysis

Mann-Whitney test was used to compare independent groups (function of subsets of CD8 T cells) or cell populations (B7-H1 and PD-1 positive cells, or apoptotic cells). The impacts of both PD-1 genotype and B7-H1 antibody on T cell apoptosis, or the therapeutic effects of different antibody were analyzed by two-way ANOVA because two independent factors (PD-1 genotype or antibody treatment) were analyzed. Comparisons of the impact of different antibody on the p38 MAPK activation were analyzed with one-way ANOVA due to the numerical independent variables. Gender and age paired groups (WT and KO mice, or T cells of the same mouse treated with or without B7-H1 antibody) were compared with 2-tailed paired t test for their different activation of p38 MAPK. All statistical analyses were performed using GraphPad Prism software 5.0 (GraphPad Software, Inc., San Diego, CA). A *P* value < 0.05 was considered statistically significant.

## Additional Information

**How to cite this article**: Liu, X. *et al*. B7-H1 antibodies lose antitumor activity due to activation of p38 MAPK that leads to apoptosis of tumor-reactive CD8^+^ T cells. *Sci. Rep.* **6**, 36722; doi: 10.1038/srep36722 (2016).

**Publisher’s note:** Springer Nature remains neutral with regard to jurisdictional claims in published maps and institutional affiliations.

## Supplementary Material

Supplementary Information
