# Supplementary material for: B7-H1 antibodies lose antitumor activity due to activation of p38 MAPK that leads to apoptosis of tumor-reactive CD8+ T cells
Source: Sci Rep. 2016 Nov 8;6:36722. doi: 10.1038/srep36722 (PMC5099859; doi:10.1038/srep36722)
Supplement: Supplementary Information [file srep36722-s1.pdf]

Supplemental information to:

B7-H1 antibodies lose antitumor activity due to activation of p38 MAPK that leads to apoptosis of tumor-reactive CD8$^+$ T cells

Xin Liu[1*], Xiaosheng Wu[2*], Siyu Cao[1], Susan M. Harrington[1], Peng Yin[1], Aaron S. Mansfield[4] and Haidong Dong[1, 3]

[1]Department of Urology, [2]Division of Hematology, [3]Department of Immunology, [4]Division of Medical Oncology, College of Medicine, Mayo Clinic, Rochester, MN 55905.

* These authors contributed equally to this work.

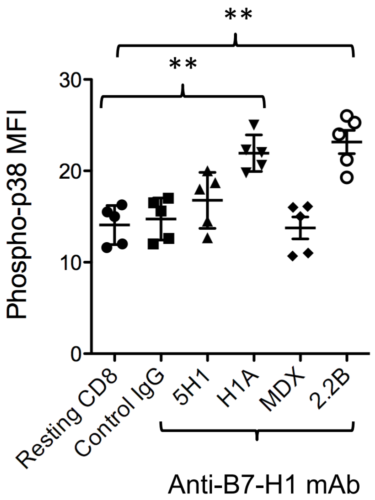

Supplemental Fig. 1. B7-H1 antibody-induced p38 MAPK activation in human primary CD8⁺ T cells. Purified human peripheral blood CD8⁺ T cells were incubated with anti-human B7-H1 mAb (clones names are shown) in the presence of anti-CD3/CD28 beads. H1A or 2.2B mAb, but not others, increased the levels (MFI) of phosphorylated p38 MAPK (phospho-p38) in activated CD8⁺ T cells. **P<0.01 (mean ± s.d., analyzed by one-way ANOVA, n=5 donors of each group).

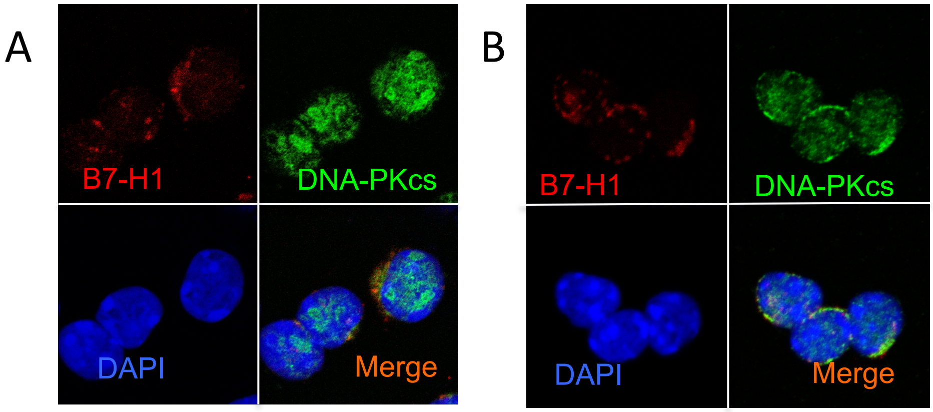

Supplemental Fig. 2. Fewer association of B7-H1 with DNA-PKcs in resting T cells. Resting $CD8^+$ T cells were isolated from spleen of naïve wild type (A) or PD-1 KO (B) C57BL/6 mice, and stained with antibodies to B7-H1 (red) and DNA-PKcs (green). Nucleus are stained with DAPI (blue).

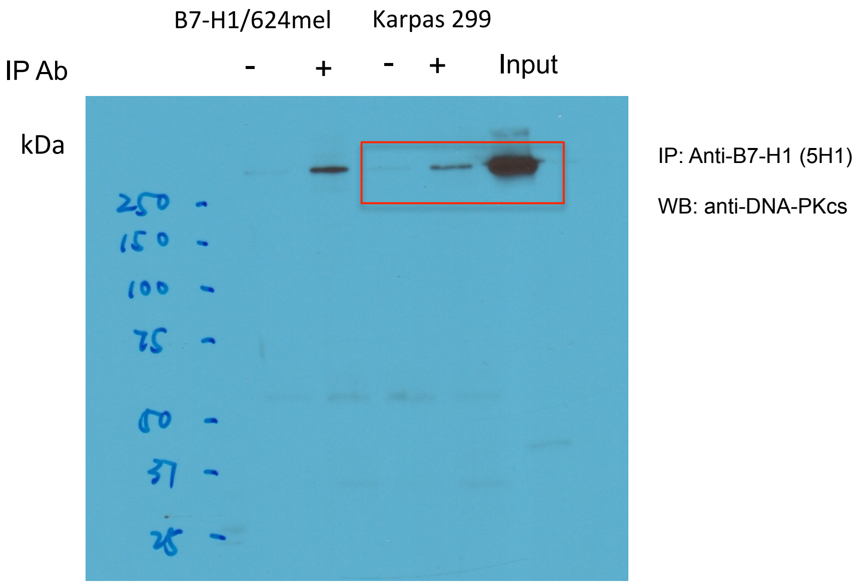

Supplemental Fig. 3. B7-H1 is associated with DNA-PKcs in human cell lines. Immunoprecipitation (IP) and Western blot (WB) identified an association of B7-H1 with DNA-PKcs in Karpas 299 cells and B7-H1 transfected melanoma cell line (B7-H1/624mel). Whole cell lysate is used as input. The association of B7-H1 with DNA-PKcs was detected by Western blotting using anti-DNA-PKcs antibody (H163). Red box indicated the location of cropped blots for presentation.

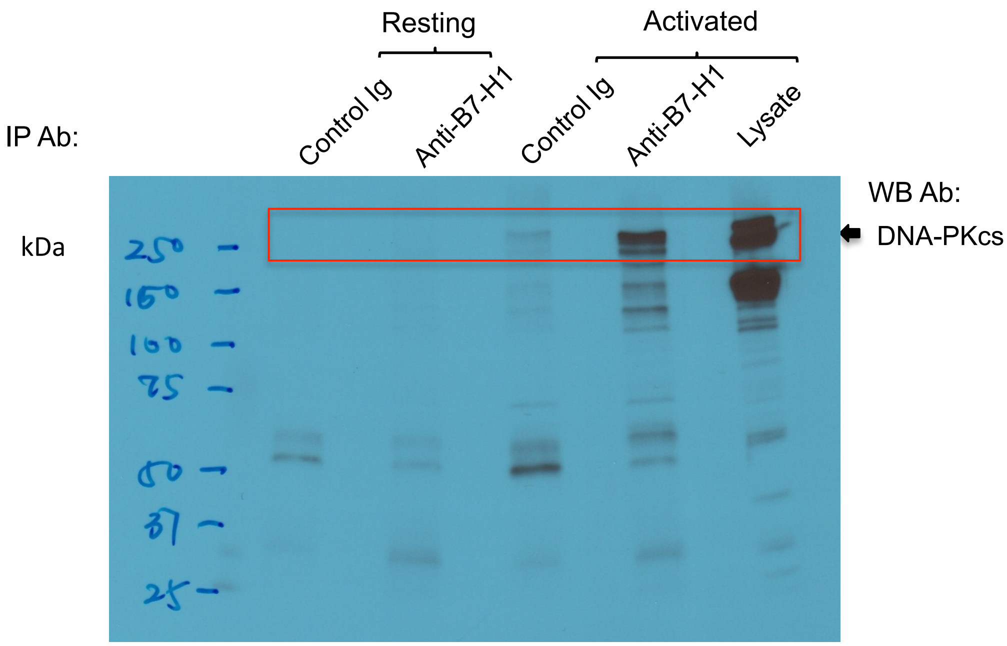

Supplemental Fig. 4. B7-H1 is associated with DNA-PKcs in human primary activated T cells. Immunoprecipitation (IP) and Western blot (WB) identified an association of B7-H1 with DNA-PKcs in primary human peripheral blood T cells that were activated with PHA incubation for 48 hours. The resting cells were not stimulated with PHA. Whole cell lysate is used as input. The association of B7-H1 with DNA-PKcs was detected by Western blotting using anti-DNA-PKcs antibody (H163). Red box indicated the location of cropped blots for presentation.
